# The Beat

**Published:** 2008-04

**Authors:** Erin E. Dooley

## Dust Up in the West

A study published in the 24 February 2008 issue of *Nature Geoscience* shows that the western United States has become 500% dustier over the past two centuries, with dust deposition higher than at any other point in the past 5,000 years. The authors based their findings on sediment records from alpine lakes in Colorado, which show that levels of nutrient and mineral deposition shot up between 1860 and 1990, coinciding with booms in mining, agriculture, ranching, and railroad activity. High dust levels can cause significant human health problems such as lung damage, allergies, and other respiratory problems. The increased levels could also have a pronounced impact on surface-water alkalinity, aquatic productivity, and nutrient cycling.

**Figure:**
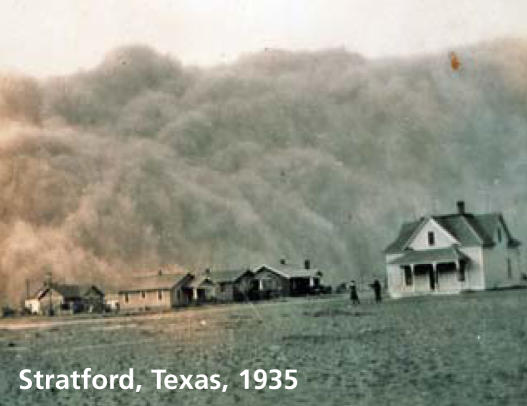
Stratford, Texas, 1935

## Tiny Particles a Great Solution?

University of South Australia researchers have discovered a new approach that could help prevent waterborne disease for millions of people. Their method, described in volume 5, issue 2/3 (2008) of the *International Journal of Nanotechnology*, is supposedly more effective and cheaper than conventional water purification technologies. The method uses silica particles covered with a nanometer-thin hydrocarbon-based coating along with an anchor that contains silicon. Stirring the particles through contaminated water for up to one hour binds pathogens, which are then filtered out along with the particles.

## Bright Nights and Breast Cancer

Using NASA satellite data showing the amount of nighttime light reaching space, scientists at the University of Haifa have shown that living in a brightly lit area may contribute to greater risk of breast cancer. The scientists generated a map of nighttime light measurements overlaid with another map showing local breast and lung cancer statistics. The scientists adjusted for such variables as smoking, income, and ethnicity. Residents of neighborhoods with the most nighttime brightness had an average 64% higher rate of breast cancer than those living in areas with the lowest nighttime light. The study appears in the January 2008 issue of *Chronobiology International*.

**Figure:**
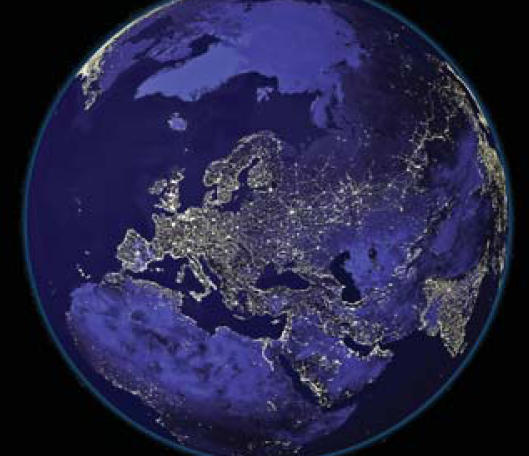


## Pollution’s Effects on Intelligence

Unlike links between air pollution and effects on the cardiovascular and respiratory systems, which have been well documented, associations between such pollution and neurologic effects remain largely unexamined. In one of the first such studies, published 1 February 2008 in the *American Journal of Epidemiology*, scientists from the Harvard School of Public Health found that children who were heavily exposed to black carbon, a component of vehicle exhaust, scored lower on several intelligence tests, with effects were similar to those seen in children whose mothers smoked 10 cigarettes a day while pregnant or who had been exposed to lead. The authors believe the effects could be the result of inflammation and oxidative damage in the brain caused by exposure to the pollutants.

**Figure:**
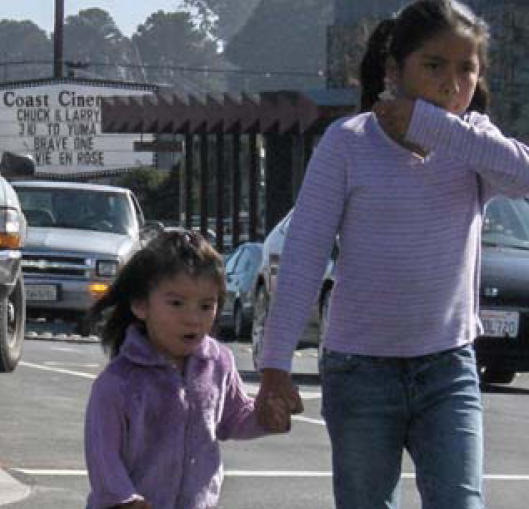


## Flour Mill Fathers Have Fewer Sons

A body of research has shown that exposures to a number of chemicals can affect men’s reproductive health as well the survival of male fetuses. Now a study in the February 2008 issue of the *American Journal of Industrial Medicine* adds to this evidence, with findings that male flour mill workers tend to father a disproportionate number of female offspring. The study also found that male offspring of these men had lower birth weights than their female counterparts. The authors believe the effects may result from the men’s occupational exposure to highly toxic pesticides used to kill insects in stored grain and flour. One such pesticide, phosphine, is a known genotoxicant. Another, 1,2-dibromo-3-chloropropane, causes testicular dysfunction and other reproductive effects.

## Nigeria Taxes e-Waste Imports

Nigeria’s information minister John Odey announced in February 2008 the Nigerian government’s plans to impose duties on imported “e-waste”—used computers, appliances, cell phones, and other electronic goods sent to developing countries for dumping or salvage. Such items are flooding Nigeria alone at rates such as 400,000 computers each month. Toxic components such as lead, cadmium, mercury, hexavalent chromium, and brominated flame retardants in the e-waste pose health threats to the people and environment of the countries where these items are shipped. Until now, imported e-waste has not been subject to duties because it has been classified as educational material.

**Figure:**
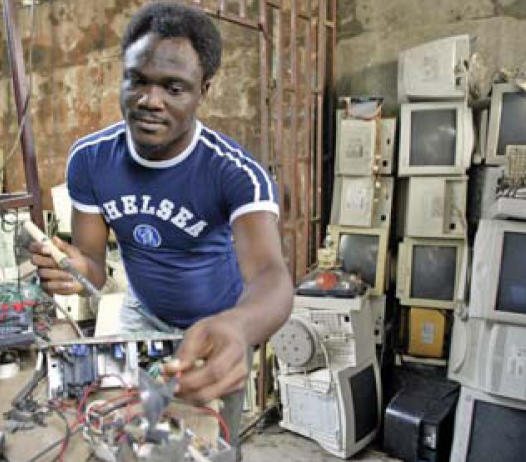
“Computer village” in Lagos, Nigeria

